# Supplementary material for: Specific PIP2 binding promotes calcium activation of TMEM16A chloride channels
Source: Commun Biol. 2021 Feb 26;4:259. doi: 10.1038/s42003-021-01782-2 (PMC7910439; doi:10.1038/s42003-021-01782-2)
Supplement: Supplementary file 2 — Supplementary Information [file 42003_2021_1782_MOESM2_ESM.pdf]

Supplementary Information

Specific PIP_2 Binding Promotes Calcium Activation of TMEM16A Chloride Channels

Zhiguang Jia[1] *and Jianhan Chen*[1,*]

[1]Department of Chemistry

and

Department of Biochemistry and Molecular Biology

University of Massachusetts

Amherst, MA 01003, USA

*Corresponding Authors: Phone: (413) 545-3386, jianhanc@umass.edu

Supplementary Tables

Table S1. Summary of atomistic simulations of TMEM16A

Systems	Ca^{2+}	PIP_2	Initial Structure	Length (μs)
sim 1-3 [1]	+	+	5oyb	sim 1: 3.0<br>sim 2,3: 1.5
sim 4-6 [1]	+	-	5oyb	sim 4: 3.0<br>sim 5,6: 1.5
sim 7-9 [1]	-	+	5oyg	1.0 each
sim 10-12 [1]	+	- (w POPS)	5oyb	0.5 each
sim 13-15 [1]	+	-(w PI(4)P)	5oyb	0.5 each
*Meta 1-4** [2]	+	+	*sim1*, 0.65 μs	0.2 Each

[1] Simulation performed with Amber

[2] Simulation performed with Gromacs

* metadynamics simulations in the Ca^{2+}/PIP_2-bound open state of TMEM16A.

Supplementary Figures

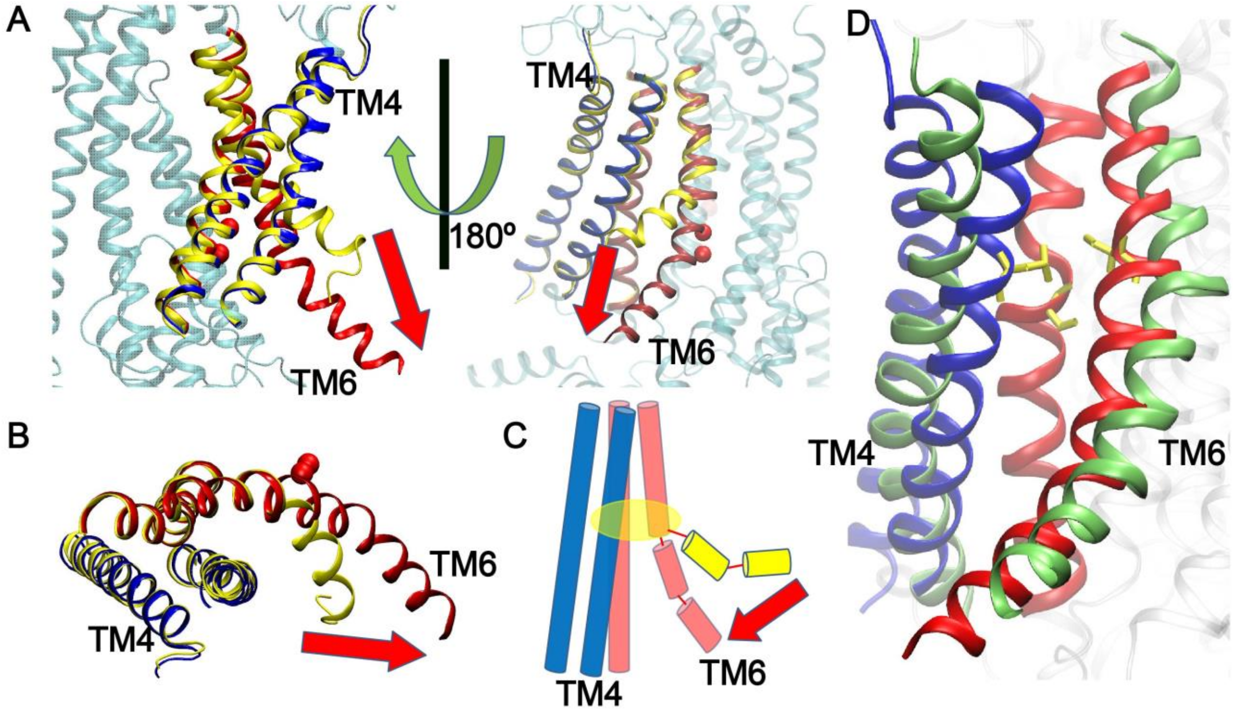

Fig. S1. Ca^{2+}-induced lower pore opening in TMEM16A. **A** and **B**, side and top views of the TMEM16A in the Ca^{2+}-free (PDB: 5oyg) and bound (PDB: 5oyb) states. TMs 3-4 and TMs 5-6 in the Ca^{2+}-bound structure are colored in blue and red, respectively; TMs 3-6 in the Ca^{2+}-free structure are colored in yellow. The movement of TM6 upon Ca^{2+} binding is highlighted with the red arrow. **C**: cartoon illustration of the movement of TM6 upon Ca^{2+} binding. **D**: Superimposed structurs of Ca^{2+}-bound TMEM16A with the fully opened nhTMEM16 lipid scamblase (PDB: 4wis). TM4 and TM6 of nhTMEM16 (green cartoons) are further separated than those of TMEM16A.

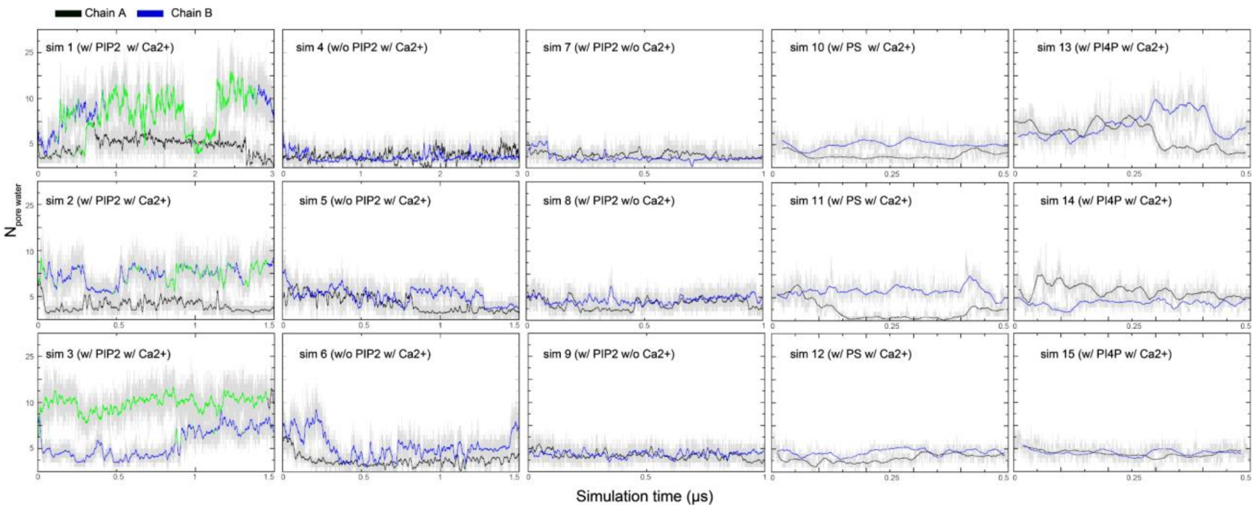

Fig. S2 Number of pore water molecules in the neck region as a function of the simulation time for Ca2+-bound TMEM16A with PIP2 (sim1-3), Ca2+-bound TMEM16A without PIP2 (sim4-6), Ca2+-free TMEM16A with PIP2 (sim7-9), Ca2+-bound TMEM16A with POPS (sim 10-12) and Ca2+-bound TMEM16A with PI(4)P (sim 13-15). All snapshots that belong to the opened state cluster (see Fig. S3) are highlighted in green. Note that TMEM16A remained in deactivated states and never sampled any open state conformation throughout all simulations without both Ca2+ and PIP2 bound (sim4-9).

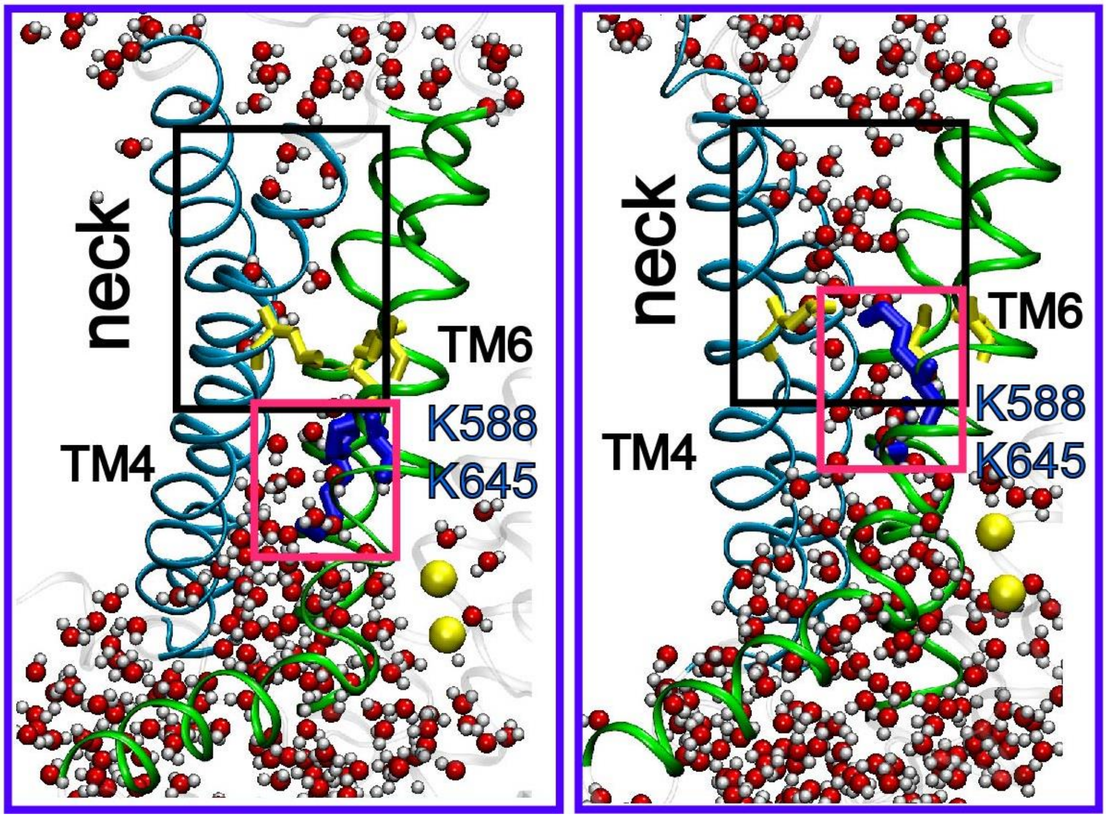

Fig. S3 Representative snapshots showing pore hydration in the collapsed state (left, Ca^{2+}-bound but without PIP_2; *sim4*, 0.535 μs) and the dilated state (right, Ca^{2+} and PIP_2 bound; *sim1*, 2.681 μs). TMs 3-4 and 5-6 are represented as cyan and green cartoons, respectively. Bound calcium ions are shown as yellow spheres and water molecule inside the pore as spheres colored by atom types (red: oxygen; white: hydrogen). The inner gate residues, L547, S592 and I641, are shown as yellow sticks. Two conserved basic residues below the gate, K588 and K645, are represented as blue sticks and highlighted with red boxes. The neck region is highlighted with a black box.

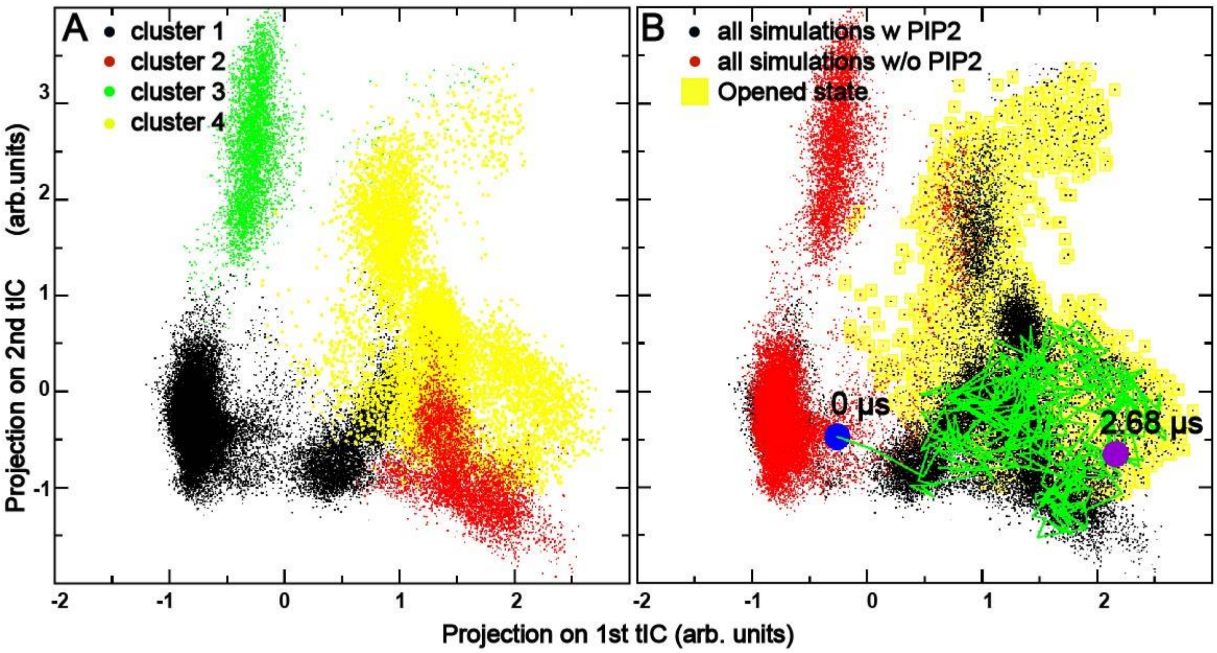

Fig. S4 Clustering of TMEM16A pore conformational space. All snapshots sampled in trajectories s*im1-6* (Ca^{2+}-bound state with and without PIP_2) were clustered based on time-lagged independent component analysis (tICA) (see Methods for details). The snapshots are projected onto the first two tICAs. **A**: The clusters are colored by cluster IDs (1-4). The cluster 4 (yellow) is the open and conductive state. Note that the clustering was performed on multi-dimensional tICA and some clusters may appear to substantially overlap in the 2D projection. **B**: The clusters are colored by simulation conditions, with snapshots from simulations of Ca^{2+}-bound TMEM16A with and without PIP2 colored in red and black, respectively. The conformational space covered by the open state cluster (cluster 4 in panel A) is marked using a yellow background. The location of the initial structure (PDB: 5oyb) is labeled using the blue circle, and the location of the selected open state pore structure (around 2.68 μs from *sim 1*, Chain B) is labeled using the purple circle. The green trace plots the trajectory of the pore structure of chain B sampled during *sim1*.

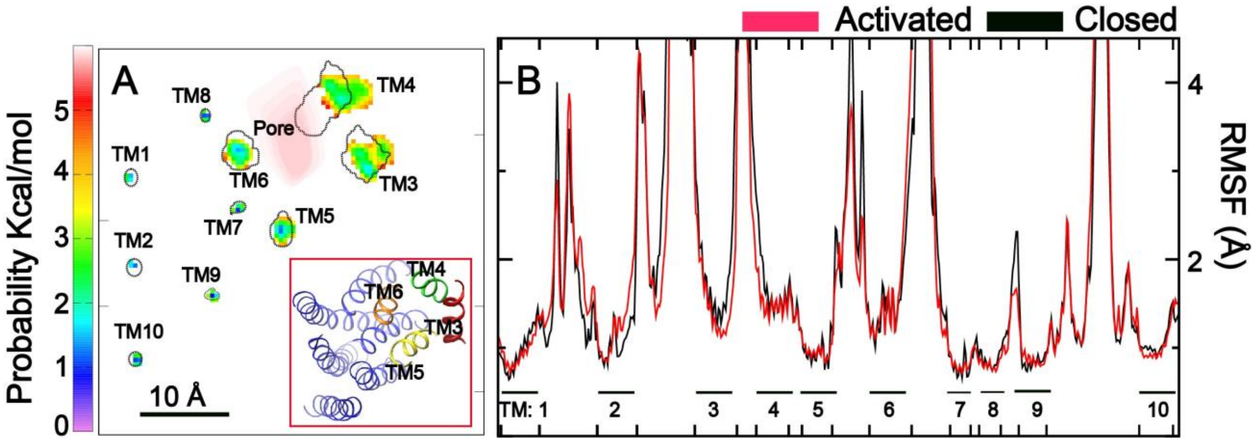

Fig. S5 Dynamics and movements of TMEM16A during activation. **A**: Distributions of the centers of mass (CMs) of TM helices along membrane lateral directions (x, y) in the closed and activated states. The distributions for the activated state are plotted as colored free energy maps , while those for the closed state are shown as the dashed contours (at level: 5 kcal/mol). For the pore-forming TMs, only upper pore segments (TM3: G510:A523; TM4: T539:L547; TM5: S592:F602 and TM6: M632:I641) are included in the CM calculation. The pore region is also indicated. **B**: Root-mean-squared fluctuation (RMSF) of backbone Cα atoms of the activated (red) and closed (black) states. The snapshots analyzed were taken from trajectories s*im1-6* (Ca^{2+}-bound state with and with PIP_2). For each simulation, both monomers are analyzed separately. All snapshots were aligned by the supporting domains (TM 1,2,7-10) before analysis. The state of each snapshots (activated or closed) was assigned based on the clustering analysis (Fig S4). The reference monomer structure used for aligning all snapshots is shown in the insert with TM3-6 colored in red, green, yellow and orange, respectively.

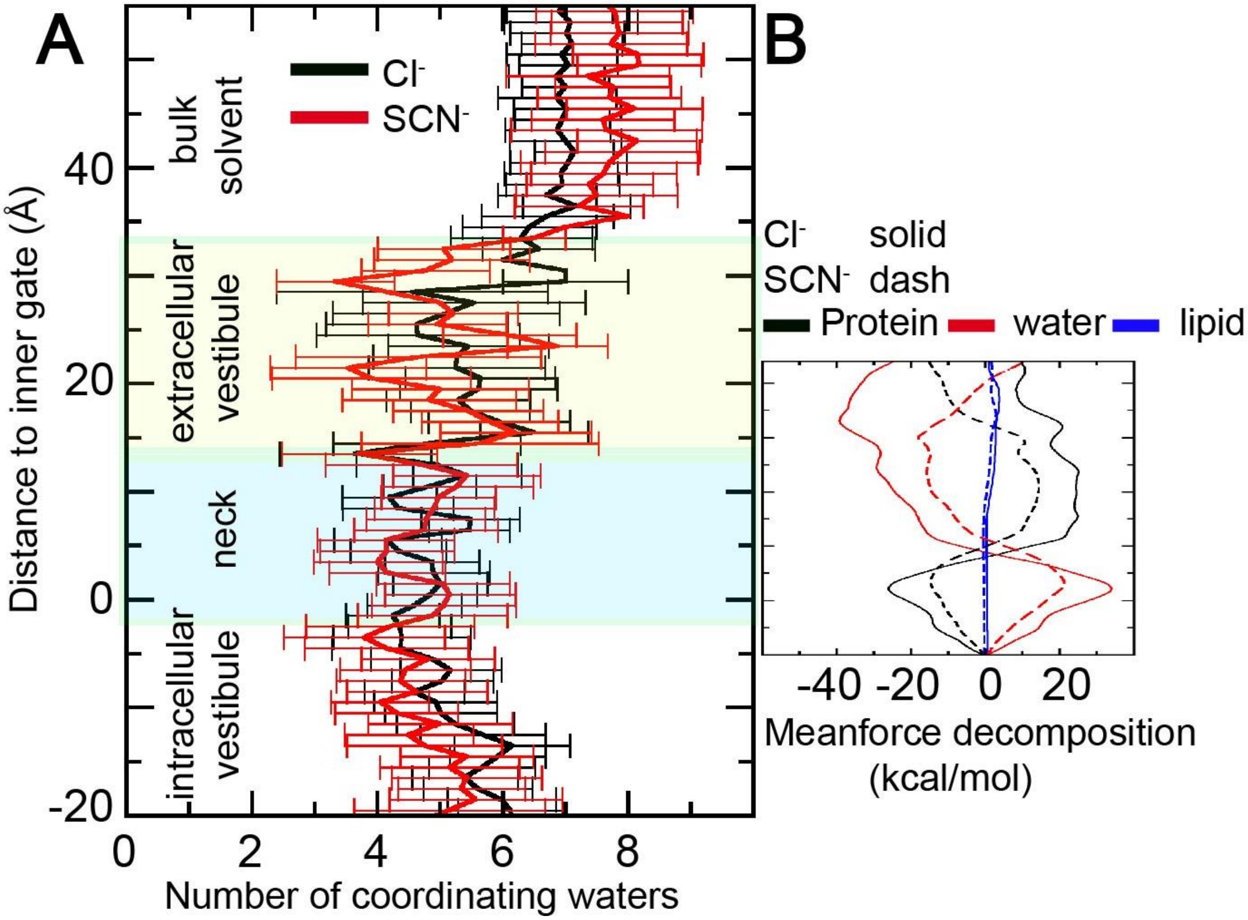

Fig. S6 Solvation property and free energy decomposition of Cl^- and SCN^- permeation. **A**. The number of solvation waters of anions inside the channel, calculated as the number of water hydrogen atoms within 2.8 Å of Cl^- (black trace) or SCN^- (red traces). The results were calculated as the average from umbrella sampling trajectories and the error bars shown are the standard deviations. **B.** Contributions of protein, water and membrane to the free energy of anion permeation calculated using mean force decomposition. Various decomposed contributions are shown in solid and dashed lines for Cl^- and SCN^-, respectively.

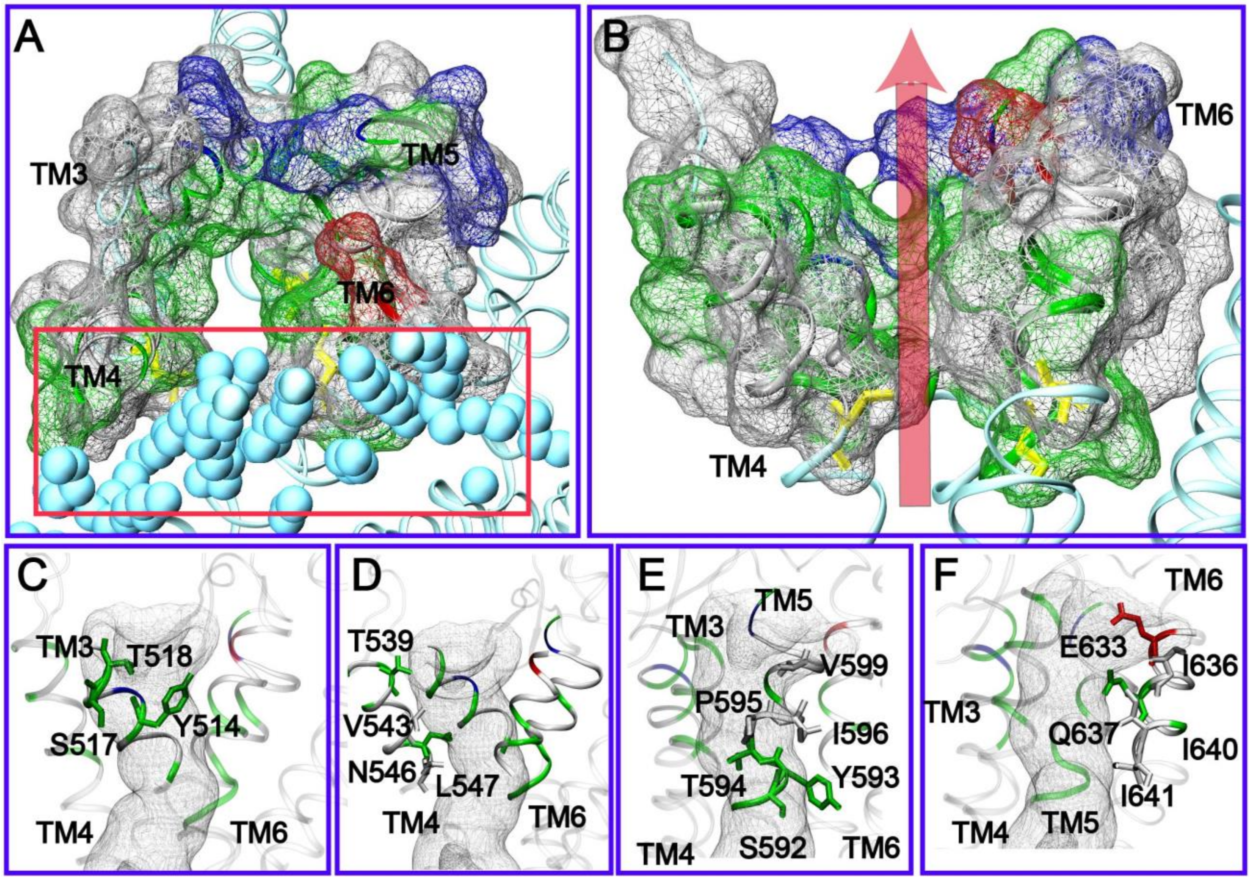

Fig. S7. Structural features of the open state of TMEM16A conduction pore. **A, B**: top and front side views of the pore surface. The van der Waals surface of the protein is represented as mesh surfaces. The hydrophobic, hydrophilic, positively charged and natively charged residues are colored in white, green, blue and red, respectively. The inner gate residues, L547, S592, I641, as yellow sticks. POPC lipid tails near TMs 4 and 6 (<9 Å heavy atom distance) are shown as cyan spheres in panels A and B. **C**-**F**: pore-lining residues on TMs 3-6, respectively. The snapshot is taken from *sim1* at 2.681 μs. The residues are colored by residue type (hydrophobic: white; hydrophilic or polar: green; negatively charged: blue; positively charged: red). The profile of the conducting pore calculated using HOLE is shown as the transparent tunnel in panels **C**-**F**.

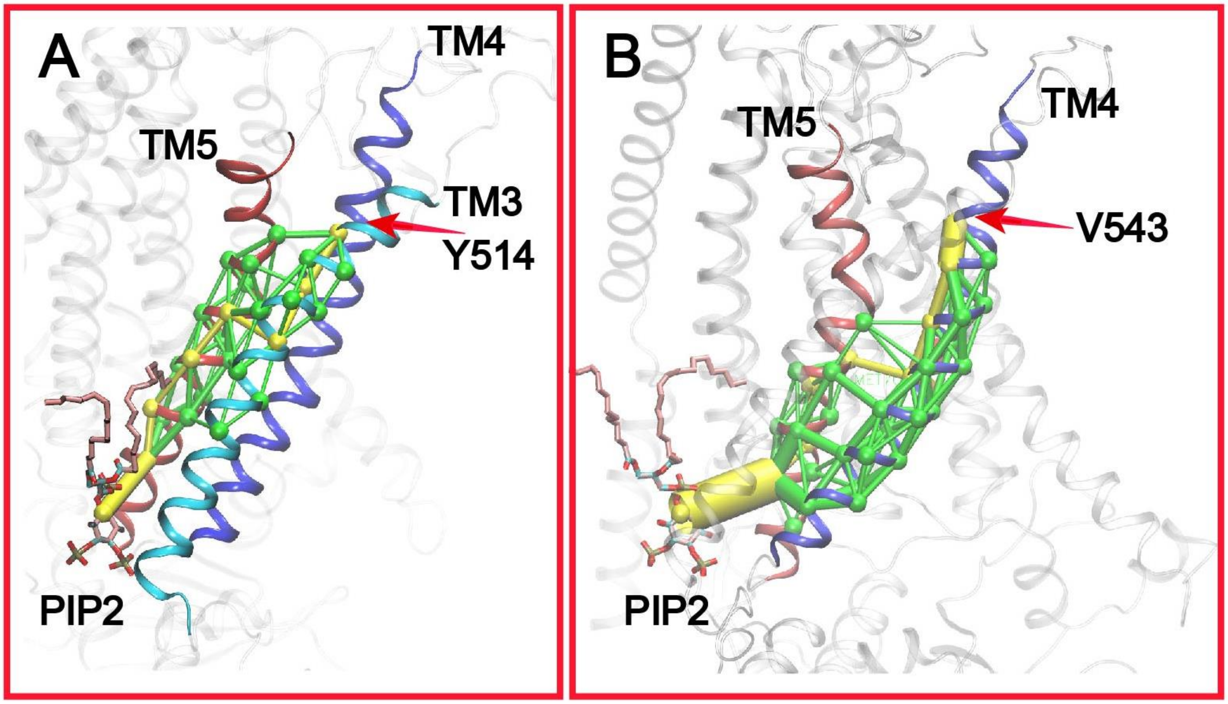

Fig. S8 Optimal and suboptimal pathways of coupling between PIP_2 and the neck region of the TMEM16A pore. **A**: Coupling pathways between PIP2 and Y514 in TM3, and **B**: between PIP_2 and V543 in TM4. TMs 3, 4 and 5 are represented as cyan, blue and red cartoons, respectively. TM3 is not colored in panel B for clarity. The residues involved in the pathway are presented as green spheres and the contacts are presented as sticks. The optimal and suboptimal pathways are colored in yellow and green, respectively. The thickness of the edges represents the number of paths crossing that edge. See Methods for details of coupling pathway analysis.

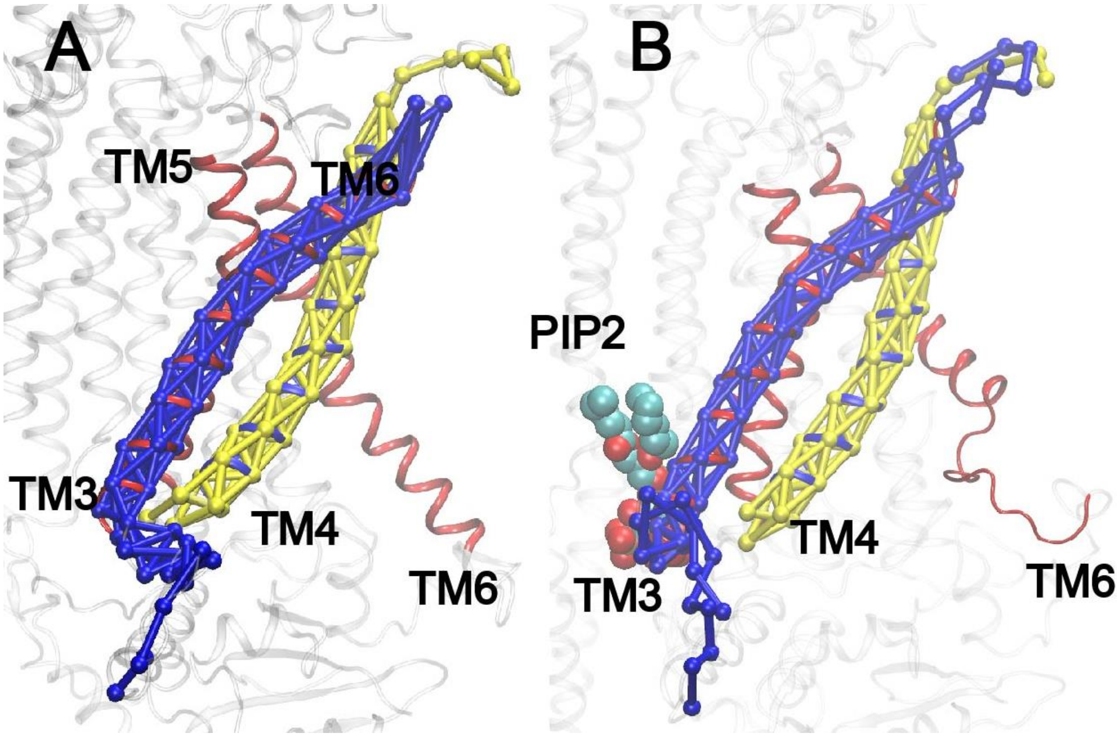

Fig. S9 Dynamic network analysis of A: Ca^{2+}-bound TMEM16A in absent of PIP_2 and B: Ca^{2+}-free TMEM16A in present of PIP_2. Result shows that TMs 3 and 4 are clustered into two large independent communities (blue and yellow networks, respectively).

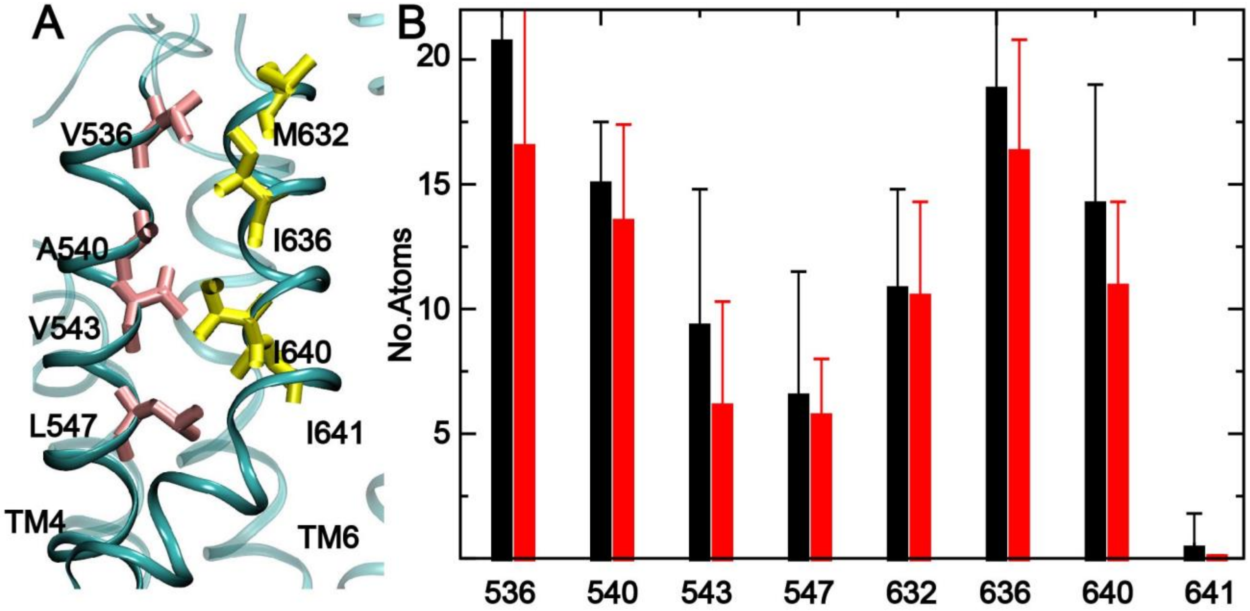

Fig. S10 A: A representative snapshot of the inactive pore in the Ca^{2+}-free state of TMEM16A (*sim 7*, 1.0 μs). The hydrophobic residues on TMs 4 and 6 are represented as pink and yellow sticks, respectively. B: The number of lipid tail heavy atoms within 5 Å of the hydrophobic residues listed in (A). The numbers for the open and closed states are colored in black and red, respectively.
