# Supplementary material for: Specific PIP2 binding promotes calcium activation of TMEM16A chloride channels
Source: Commun Biol. 2021 Feb 26;4:259. doi: 10.1038/s42003-021-01782-2 (PMC7910439; doi:10.1038/s42003-021-01782-2)
Supplement: Supplementary file 3 — Description of Additional Supplementary Files [file 42003_2021_1782_MOESM3_ESM.pdf]

Description of Additional Supplementary Files

File name: Supplementary Movie 1

Description: Spontaneous opening of the Ca2+-bound TMEM16A pore induced by specific PIP2 binding. The movie was generated based on sim1. Only chain B is shown in cartoons, with TMs 3-4 colored in blue, TMs 5-6 in red and the rest in light green. The phosphate atoms in lipid head groups are represented as transparent spheres, and water molecules near the pore shown in sticks. The bound PIP2 molecule is shown in ball-and-stick. The two bound Ca2+ ions are shown as two red spheres. The inner gate residues, L547, S592 and I641, are shown as yellow sticks. Only water molecules near the pore are shown for clarity.

File name: Supplementary Movie 2

Description: The Ca2+-bound TMEM16A pore remained collapsed without PIP2. The movie was generated based in Chain B from sim4. See the Movie S1 caption for details of molecular rendering. Only water molecules near the pore are shown for clarity.

File name: Supplementary Movie 3

Description: The Ca2+-free TMEM16A pore remained collapsed even with specific binding of PIP2. The movie was generated based in Chain A from sim7. See the Movie S1 caption for details of molecular rendering.

File name: Supplementary Movie 4

Description: Spontaneous Cl- permeation through the open pore of TMEM16A. The movie was generated based on sim3. The permeating chloride is shown as a red sphere with

coordinating waters shown in sticks. See the Movie S1 caption for details of molecular rendering. Only the ion coordination water molecules are shown for clarity.

File name: Supplementary Data 1

Description: The data plotted in Fig. 2, 3 and 4.

File name: Supplementary Data 2

Description: Snapshot of TMEM16A in opened state taken from sim 1 (Ca^{2+}-bound TMEM16A with PIP2).

File name: Supplementary Data 3

Description: Snapshot of TMEM16A in closed state taken from sim 4 (Ca^{2+}-bound TMEM16A without PIP2).
